# Mental imagery and performance in aerobic, artistic, acrobatics, trampoline and tumbling, and rhythmic gymnasts: a systematic review

**DOI:** 10.3389/fpsyg.2026.1803261

**Published:** 2026-03-11

**Authors:** Wenxin Yang

**Affiliations:** Chengdu Sport University, Chengdu, China

**Keywords:** gymnastics performance, mental imagery, motor learning, psychological skills training, sports performance

## Abstract

**Objectives:**

To identify and synthesize evidence on mental imagery (and imagery-based methods) in competitive gymnasts, evaluate effects on performance and relevant psychological/psychophysiological correlates, and appraise methodological quality and important intervention features.

**Methods:**

PubMed, Scopus, and Web of Science Core Collection were searched. Eligible studies included competitive gymnasts from any FIG discipline examining imagery as an intervention/exposure or as an imagery construct associated with performance-relevant outcomes. Risk of bias was assessed using RoB 2 for randomized studies and ROBINS-I for non-randomized/observational. Results were synthesized narratively by study design, imagery approach, and outcome domain.

**Results:**

Searches yielded 393 records; 258 unique records were screened; 46 full texts were assessed; and 16 studies were included. Interventions varied (script-based imagery, PETTLEP-informed imagery, video observation plus imagery, and multi-component psychological skills training). Several controlled/quasi-controlled studies reported improved gymnastics-related performance outcomes (judged skill execution or sport-specific performance indices) with imagery-based approaches, whereas others found no performance benefit despite improvements in psychological variables (e.g., self-confidence). Effects appeared moderated by expertise level, sequencing/dose, and outcome choice. Across RoB 2 studies, 0/8 (0%) were overall low risk, 4/8 (50%) had some concerns, and 4/8 (50%) were high risk, with the most frequent domain drivers being randomization process, outcome measurement, and selective reporting. Across ROBINS-I studies, 2/8 (25%) were moderate, 5/8 (62.5%) serious, and 1/8 (12.5%) critical, driven mainly by confounding, then outcome measurement and selective reporting.

**Conclusion:**

Imagery is a potentially useful adjunct to gymnastics training, but effects are inconsistent and implementation- and athlete-dependent. Higher-quality, transparently reported trials using standardized, competition-relevant outcomes are needed.

**Systematic review registration:**

Open Science Framework (osf/io/a9tj6; Date: 25/01/2026).

## 1 Introduction

In judged gymnastics disciplines, final rankings are shaped not only by athletes’ technical execution but also by properties of evaluation systems, including potential variability and bias in judging (Fung et al., 2025). Empirical analyses in rhythmic gymnastics show that the quality and consistency of difficulty judging can vary across competitive levels, illustrating that evaluation reliability is a nontrivial factor in performance outcomes (Leandro et al., 2017). In artistic gymnastics, continuous monitoring during competition has linked higher perceived nervousness to lower routine performance on balance beam, reinforcing the sensitivity of complex routines to acute competitive anxiety (Cottyn et al., 2006). Across competitive gymnasts, injury burden has been associated with higher anxiety and adverse mood states, suggesting bidirectional interactions between physical stress and psychological readiness (Kolt and Kirkby, 1994). These findings are consistent with broader evidence that anxiety–performance relationships are heterogeneous and athlete-specific, which makes targeted psychological skill development particularly relevant in high-pressure, precision-based sports (Raglin, 1992).

Mental imagery in sport involves the deliberate creation or re-creation of sensory experiences in the absence of external input and has been theorized to influence performance through both cognitive and motivational functions (Paivio, 1985). Within applied sport models, imagery use is often distinguished by its function (e.g., cognitive-specific rehearsal of technique versus motivational-general mastery/affect regulation) and by its content (e.g., process-focused imagery emphasizing movement execution cues versus outcome-focused imagery emphasizing scores or competitive results), distinctions that are directly relevant to the heterogeneous gymnastics interventions (Paivio, 1985). Foundational work on mental practice further indicates that imagery effectiveness depends on image quality (e.g., vividness and controllability), task alignment, and individual differences in imagery capacity (Denis, 1985). Accordingly, imagery quality should be treated as a multidimensional construct that can be operationalized via validated psychometrics and/or via session-level manipulation checks (e.g., vividness/ease ratings, adherence to prescribed perspective/timing), because inadequate fidelity can plausibly attenuate effects even when imagery is prescribed (Suica et al., 2022). Neuroimaging meta-analytic evidence supports substantial overlap between networks engaged during motor imagery, action observation, and movement execution, providing a mechanistic basis for imagery-based rehearsal of motor skills (Hardwick et al., 2018). Experimental neurophysiology likewise shows that motor imagery can generate brain activity patterns paralleling those observed during motor execution, which supports mechanistic plausibility but does not, by itself, establish training efficacy for gymnastics performance outcomes (Kraeutner et al., 2014). However, systematic evidence also emphasizes that imagery outcomes depend on how imagery is implemented (e.g., timing, perspective, and integration with physical practice), with substantial heterogeneity across tasks and performer characteristics (Schuster et al., 2011). For example, gymnasts often exhibit strong kinesthetic mental imagery characteristics and distinct sensorimotor signatures during mental imagery tasks, however, such differences may reflect a combination of training-related neuroplasticity and pre-existing characteristics that influence self-selection into, and persistence within, high-skill gymnastics pathways (Sugino and Ushiyama, 2021).

Across sport and exercise contexts, multilevel meta-analytic evidence indicates that imagery practice can enhance athletic performance across multiple outcome domains, although effects vary with protocol design and context (Liu et al., 2025). Consistent with this view, a recent Bayesian multilevel meta-analysis of athletes reported a small-to-moderate overall benefit of imagery practice on athletic performance (Liu et al., 2025). The same synthesis indicated that effects were outcome-dependent and that training dose moderated outcomes, with comparatively stronger effects reported for protocols approximating ~10 min per session, ~3 sessions/week, and longer intervention durations (e.g., ~100 days) (Liu et al., 2025).

In strength-related outcomes specifically, systematic review evidence suggests that mental imagery can improve muscular strength and that effects may be moderated by imagery modality (e.g., internal vs. external) and psychosocial factors such as self-efficacy and motivation (Slimani et al., 2016). In gymnastics-type motor tasks, imagery perspective has been shown to differentially affect learning and performance, implying practical consequences for how gymnasts mentally represent and rehearse complex actions (White and Hardy, 1995). In national-level rhythmic gymnasts, a structured intervention combining video observation with motor imagery improved jumping performance, demonstrating feasibility and performance relevance of imagery-based methods in aesthetic–technical sports (Battaglia et al., 2014). Neurocognitive evidence further suggests that gymnasts can show enhanced sensorimotor modulation during kinesthetic motor imagery compared with non-gymnasts, consistent with the sport’s reliance on refined internal movement representations (Sugino and Ushiyama, 2021). Nonetheless, imagery effects are not uniform, and individual imagery ability can moderate learning benefits from related cognitive techniques, reinforcing the need to consider athlete-specific responsiveness when translating protocols to new disciplines (Lawrence et al., 2013).

Despite a growing gymnastics literature describing training-related adaptations and performance-relevant physical characteristics, much of this work is centered on conditioning and routine-related physical outcomes rather than on psychological skill acquisition (Kyselovičová and Zemková, 2024). In contrast, psychological research in gymnastics has more frequently targeted artistic and rhythmic disciplines, including competition anxiety during routines and its association with performance (Cottyn et al., 2006). This matters for an imagery-focused synthesis because imagery interventions may plausibly influence execution consistency under pressure and fatigue and routine-level outcomes that are ultimately filtered through judging processes. At the intervention level, best-practice syntheses emphasize that imagery content, delivery parameters, and integration with physical practice shape effectiveness, which limits straightforward generalization across disciplines with distinct movement structures and competitive demands (Schuster et al., 2011). However, evidence indicates that imagery perspective effects are task-dependent, for instance, internal vs. external visual imagery can differentially influence learning and retention, including findings in a gymnastics-type task where external visual imagery showed advantages under some conditions (White and Hardy, 1995). In gymnastics, where athletes must repeatedly manage multi-axis rotations, inversion, and rapid spatial reorientation, perspective selection may plausibly interact with vestibular–multisensory processes supporting first-person perspective, self-location, and mental spatial transformation (Pfeiffer et al., 2014).

Additionally, because imagery ability varies and can influence the magnitude of learning and performance benefits from cognitively based training, discipline-specific evidence is needed to guide screening, prescription, and implementation in applied contexts (Suica et al., 2022). In competitive gymnastics, this discipline-specificity is especially pronounced because performance is ultimately operationalized through judge-derived scores, and empirical work in artistic and rhythmic gymnastics shows that reliability/validity and bias-related properties of judging can meaningfully shape observed performance outcomes (Bučar Pajek et al., 2013). Gymnastics skills also impose high coordinative and perceptual–motor complexity (e.g., multi-axis rotations, inversion, and rapid spatial reorientation), which strengthens the rationale for synthesizing imagery evidence around movement-representation demands that may differ from many non-judged, less acrobatic sports (Heinen et al., 2012). Accordingly, the unique combination of judged evaluation, exceptional coordinative complexity, and injury-related constraints motivates a gymnastics-specific synthesis that foregrounds outcome validity, task representational fidelity (complex acrobatic coordination), and implementability under restricted practice conditions, rather than a generic imagery in sport narrative. A systematic review focusing on mental imagery and performance in competitive gymnasts is therefore timely to consolidate the evidence base, evaluate methodological quality, and inform evidence-based recommendations for training and competition preparation. Accordingly, this systematic review aims to: (1) identify and synthesize studies examining mental imagery (and related imagery-based methods) in competitive gymnasts; (2) evaluate the effects of imagery on performance outcomes and relevant psychological or psychophysiological correlates; and (3) appraise methodological quality and characterize key intervention features (e.g., imagery modality, perspective, dosage, delivery format) to inform best-practice recommendations and future research directions. To organize the present review, we adopt a logic model in which mental imagery intervention features shape proximal mechanisms, which in turn influence gymnastics-relevant outcomes spanning isolated skill elements, routine execution quality, and competition performance. This scheme aligns with evidence that mental imagery engages a distributed action network overlapping with movement execution and that training can modify the structure and functional utility of mental representations supporting skilled action. Accordingly, we use this framework to structure synthesis across mental imagery ability and expertise, mental imagery delivery characteristics (including perspective and modality), and mental imagery performance endpoints that map onto gymnastics’ technical and evaluative demands.

## 2 Methods

This systematic review followed the standards and recommendations of PRISMA for reporting systematic reviews. Moreover, the systematic review protocol was registered *a priori* at Open Science Framework (osf/io/a9tj6; Date: 25/01/2026). No modifications to the protocol were executed.

### 2.1 Eligibility criteria

Studies were eligible if they included competitive gymnasts indexed by The International Gymnastics Federation, regardless of discipline (any sex, age group, or competitive level), and examined mental imagery or imagery-based psychological skills (e.g., motor imagery, mental practice, imagery rehearsal, PETTLEP-informed imagery, or combined video observation plus imagery), either as an intervention/exposure or as a measured psychological construct associated with performance. Studies were required to report at least one performance-relevant outcome, defined as competitive performance indicators (e.g., judged routine scores), objective sport-specific physical or technical performance tests, or validated task-performance measures directly relevant to competitive gymnastics. Eligible study designs included randomized and non-randomized intervention studies, quasi-experimental studies, and observational (cross-sectional or prospective) studies, provided they involved competitive gymnasts and reported eligible outcomes. Studies were excluded if participants were not competitive gymnasts (e.g., students or physical education cohorts) or if the report did not include an imagery construct or an imagery-based method. Reviews, editorials, commentaries, protocols without results, and single-case reports were excluded. No restrictions were applied on publication year. Reports were considered regardless of language when a translation sufficient for eligibility assessment and data extraction could be obtained. For studies included after full-text assessment, data extraction was performed using either professional translation (full text or targeted results/methods sections) or extraction from the original language by a fluent reader.

### 2.2 Information sources

A systematic search was conducted in PubMed, Scopus, and Web of Science Core Collection on January 27, 2026. In addition to database searches, the reference lists of all included studies and of closely related reviews located during screening were examined to identify additional eligible reports. All sources were considered searched as of January 27, 2026, and this date was reported as the final search date for each information source.

### 2.3 Search strategy

Search strategies were developed to capture the intersection of competitive gymnastics disciplines and mental imagery constructs, combining controlled vocabulary (where applicable) with free-text keywords and synonyms for imagery (e.g., “mental imagery,” “motor imagery,” “imagery rehearsal,” “mental practice”) and for the sport. The full, reproducible search strings is presented in Table 1.

**Table 1:** Search strategy.

Domains	Search specificities	Search terms
Competitive gymnastics	Title, abstract and keywords (topic)	“aerobic gymnastics” OR “aerobic gymnasts” OR “sport aerobics” OR “competitive aerobics” OR “gymnastics” OR “gymnasts” OR “artistic gymnastics” OR “acrobatic gymnastics” OR “acrobatics” OR “trampoline gymnastics” OR “tumbling” OR “power tumbling” OR “tumbling gymnastics” OR “rhythmic gymnastics” OR “rhythmic sportive gymnastics”
		AND
Mental imagery	Title, abstract and keywords (topic)	“mental imagery” OR imagery OR “sport imagery” OR “athletic imagery” OR “performance imagery” OR visualization OR visualisation OR “motor imagery” OR “movement imagery” OR “action imagery” OR “kinesthetic imagery” OR “kinaesthetic imagery” OR “visual imagery” OR “mental practice” OR “mental rehearsal” OR “imagery rehearsal” OR “symbolic rehearsal” OR “covert practice” OR “covert rehearsal” OR “motor simulation” OR “mental simulation” OR “action simulation” OR PETTLEP OR “guided imagery” OR “imagery training” OR “imagery intervention”

### 2.4 Data collection

Data were extracted by the author using a standardized, piloted extraction form developed for this review. To strengthen methodological approach, an external expert (blinded to the review objectives and study hypotheses) independently extracted data in parallel using the same form. The two extractions were cross-checked, and any discrepancies were resolved by consensus between the author and the blinded external expert, with adjudication by a second independent blinded expert when necessary. When multiple reports corresponded to the same underlying study, data were collated at the study level and the most complete dataset for each outcome/time point was retained. Inconsistencies were resolved by comparing reports and prioritizing the most detailed or most recent source. If required data were missing or ambiguously reported, study investigators were contacted to request clarification or additional information.

### 2.5 Data items

The primary outcome domain for this review was performance in competitive gymnastics, operationalized as competition-related performance indicators (e.g., judged scores) and/or objective sport-relevant performance measures (e.g., technical execution metrics, routine components, or validated discipline-specific tests), collected at all reported time points. We conceptualized performance *a priori* as a hierarchy of outcome levels reflecting increasing distance from real-world competitive scoring, namely competition routine performance, routine-component or element/skill performance, objective technical execution measures, and proxy biomechanical/physical markers plausibly related to gymnastics performance. Secondary outcome domains included psychological and psychophysiological correlates plausibly related to performance and imagery use, such as imagery ability/quality, competitive anxiety, confidence/self-efficacy, attentional measures, and related constructs when reported alongside performance outcomes. We specified an a priori logic model in which imagery exposure/intervention characteristics (content, perspective, modality, timing, and dose) are expected to influence self-regulation and arousal processes, and movement representation processes, which together are hypothesized to shape execution quality and, ultimately, competition-relevant performance outcomes.

All results compatible with each eligible outcome domain were sought. When studies reported multiple measures within the same domain (e.g., multiple performance tests or multiple judged components), all were extracted and later organized by domain and measurement context to preserve interpretability. Additional variables extracted included participant characteristics (sample size, age, sex, competitive level, training background), study design and context, imagery exposure/intervention characteristics (type, theoretical framework when stated, perspective, modality, dosage/frequency/duration, delivery format, integration with physical practice, and co-interventions), and comparator conditions where applicable. *A priori*, imagery exposures/interventions were coded into mutually exclusive delivery categories to improve interpretability and to avoid an undifferentiated imagery in sport description: (i) imagery-only (imagery/mental practice as the sole added component), (ii) imagery + modeling/observation (e.g., video/self-modeling or observation explicitly paired with imagery), and (iii) multi-component psychological package including imagery (imagery delivered alongside additional psychological skills such as relaxation, self-talk, goal setting, or attentional training).

### 2.6 Risk of bias assessment

Risk of bias was assessed at the study level by the author and an independent external expert, working in parallel and using prespecified criteria. Agreement between both was tested and was observed to be *k* = 0.94. Disagreements were resolved through discussion and, when required, adjudication by a second independent external expert. Randomized controlled trials were assessed using RoB 2, and non-randomized intervention studies were assessed using ROBINS-I. For individually randomized parallel trials, RoB 2 domain judgments (Domains [D] 1–5) followed the signaling-question logic, with D1 rated ‘high risk’ when sequence generation/allocation concealment was absent/clearly compromised, and D4 upgraded to ‘some concerns/high risk’ when outcomes were judge-scored without explicit assessor blinding or when differential measurement was plausible. For crossover trials, we additionally evaluated bias from period and carryover effects by checking for adequate washout, counterbalancing, prespecified handling of period effects, and analyses appropriate for within-person comparisons. When carryover/period effects could plausibly contaminate estimates or were not addressed, the relevant RoB 2 judgments were conservatively increased (typically impacting D2 and/or D4 and the overall rating).

### 2.7 Data synthesis

Because the review aimed to summarize evidence on imagery–performance relationships and imagery-based interventions in competitive gymnasts, effect estimates were extracted as reported in each study. For intervention studies, between-group differences (e.g., mean differences, standardized mean differences) and within-group changes were recorded when available, along with corresponding measures of precision (e.g., confidence intervals, standard errors, or *p*-values). For observational studies, association measures (e.g., correlation coefficients, standardized regression coefficients, odds ratios where applicable) were extracted with their reported precision or significance indicators.

Studies were first organized into predefined synthesis groupings based on study design and the role of imagery, distinguishing imagery-based interventions from observational studies assessing imagery constructs and their relationship to performance outcomes. However, because imagery research in gymnastics is conceptually heterogeneous (intervention content, delivery, outcomes, and athlete/context factors), synthesis was additionally conducted as a framework-based analysis. An *a priori* framework was used to code each study into contexts, mechanisms, and outcomes. Within these groupings, studies were further categorized by imagery type (e.g., motor imagery/mental practice, PETTLEP-informed imagery, combined video plus imagery), by outcome domain (competition scores, technical/skill metrics, discipline-specific performance tests), and by participant characteristics (age category and competitive level) when sufficient information was available.

Data were prepared for synthesis by harmonizing outcome descriptions, standardizing terminology for imagery modalities/perspectives, and converting reported statistics to comparable effect measures only when the underlying data and assumptions were clearly supported by the study report. Results of individual studies were presented in structured tables summarizing study design, sample characteristics, imagery exposure/intervention details, outcome measures, and key findings, and were accompanied by a textual synthesis emphasizing direction and consistency of effects and the credibility of the underlying evidence. Where patterns of variation across studies were apparent, heterogeneity was explored descriptively by comparing results across the prespecified groupings (e.g., intervention versus observational evidence, imagery perspective/modality, and competitive level).

## 3 Results

### 3.1 Study selection

**Figure 1:**
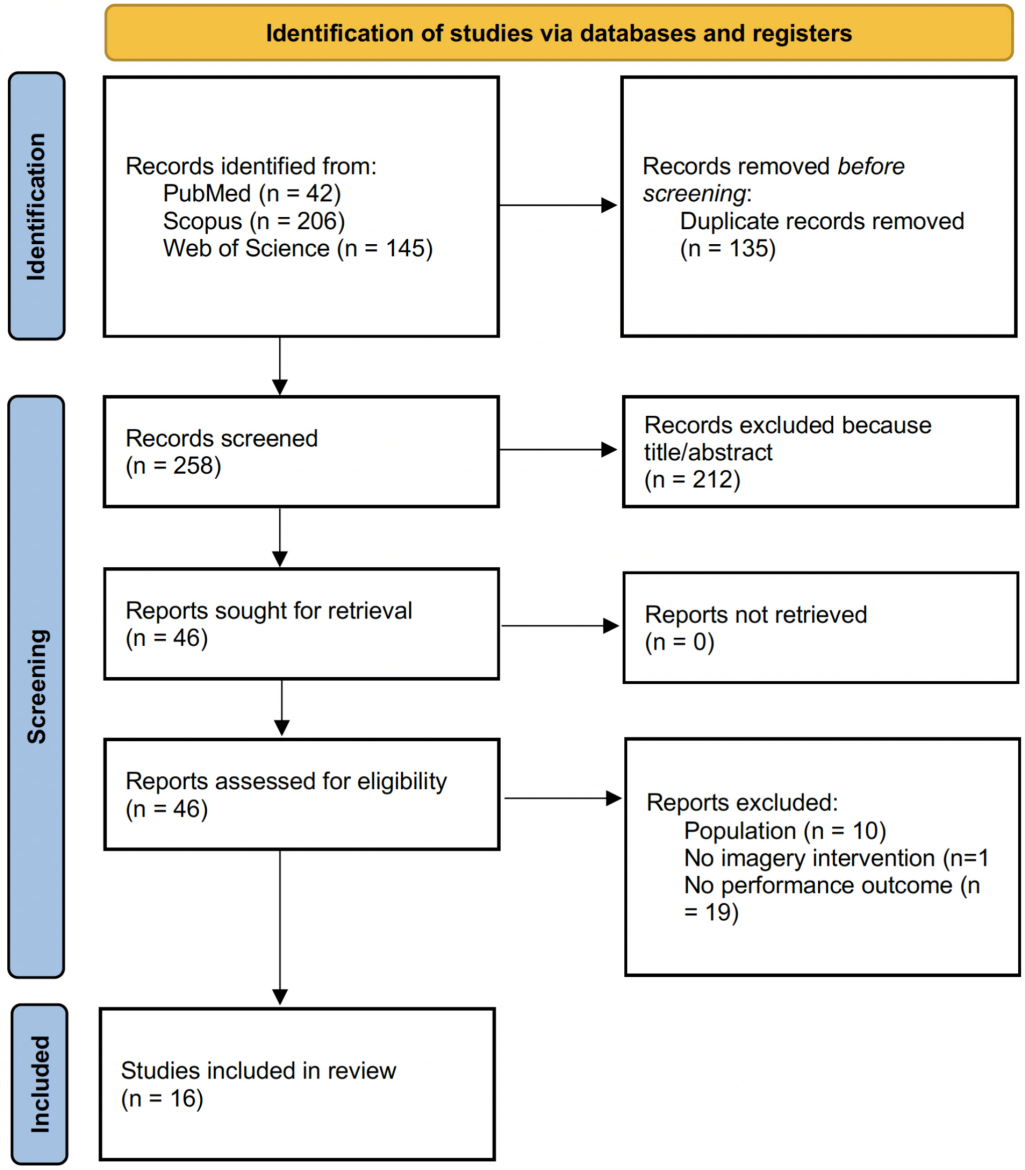
PRISMA flow diagram.

Figure 1 depicts the PRISMA 2020 flow diagram detailing how studies were located, screened, and ultimately included. Electronic searches retrieved 393 records in total (PubMed, *n* = 42; Scopus, *n* = 206; Web of Science, *n* = 145). After removing 135 duplicates, 258 unique records underwent title/abstract screening, during which 212 were excluded. The full texts of 46 reports were then requested and all were obtained and assessed for eligibility. Following full-text evaluation, 30 reports were excluded because the population did not meet inclusion criteria (*n* = 10) (Ferreira et al., 2021; Hardy and Callow, 1999; Lawrence et al., 2013; Mace and Carroll, 1989; Munzert et al., 2008; Prastyawan et al., 2023; Rohleder and Vogt, 2018; Šešum and Kajtna, 2018; White and Hardy, 1995; Yuzela et al., 2023), the study did not include an imagery intervention (*n* = 1) (Irwin et al., 2020), or no relevant performance outcome was reported (*n* = 19) (Artlip et al., 2022; Clowes and Knowles, 2013; Di Tella and Santarcangelo, 2025; Fluthmann et al., 2023; Habacha, 2019; Habacha et al., 2014; Hars and Calmels, 2007; Ille and Cadopi, 1999; Khalfallah et al., 2022; Klotzbier and Schott, 2024; Laureys et al., 2024; Liggett and Hamada, 1993; Raiola et al., 2013; Schmidt et al., 2016; Simpson et al., 2020; Sugino and Ushiyama, 2021; Tóth-Hosnyanszki et al., 2023; Veit and Vogt, 2024; White and Hardy, 1998). Overall, only 16 studies satisfied the eligibility criteria and were included in the review, highlighting a sparse and fragmented evidence base distributed across multiple gymnastics disciplines, heterogeneous tasks/outcomes, and markedly different imagery exposures and intervention configurations, limitations that constrain cross-study comparability and the strength of synthesis.

### 3.2 Study characteristics

Supplementary Table 1 summarizes the methodological and population characteristics of the included studies to contextualize the evidence base. It presents, for each study, the study design, The International Gymnastics Federation discipline and task, competitive level, sample size and group structure, participant demographics (age and sex where reported), the primary performance outcomes and any secondary outcomes measured, and the measurement timepoints. Artistic gymnastics dominates (12/16; 75%), while rhythmic (2/16), aerobic (1/16), and acrobatic (1/16) are minimally represented; several FIG disciplines are absent entirely from the included-study set. Across age coverage, adult-only samples are rare (1/16; 6.25%) and many studies either focus on youth or span youth–adult ranges, and performance outcomes cluster mainly around component-level metrics and proxies, with only 3/16 (18.75%) using routine/competition-aligned judged endpoints.

Supplementary Table 2 details the characteristics of the mental imagery intervention procedures in the included studies. For each study, it specifies the imagery type and content/function, imagery perspective and modality, timing/context of delivery, dosage, delivery format, and how imagery was integrated with physical practice, alongside co-interventions, comparator/control conditions, and any manipulation checks or imagery ability measures used.

### 3.3 Risk of bias assessment

Table 2 summarizes the risk of bias for studies using randomized designs (including crossover/cluster-randomized formats where applicable) evaluated with the Cochrane RoB 2 tool.

**Table 2:** Risk of bias in randomized studies.

Study	D1 randomization process	D2 deviations from intended interventions	D3 Missing outcome data	D4 Measurement of the outcome	D5 Selection of the reported result	Overall risk of bias
Battaglia et al. (2014)	Low risk	Low risk	Low risk	Low risk	Some concerns	Some concerns
Guo (2014)	Some concerns	Some concerns	No information	High risk	Some concerns	High risk
Khalaf et al. (2024)	High risk	High risk	No information	Some concerns	Some concerns	High risk
Marshall and Gibson (2017)	Some concerns	Some concerns	Some concerns	High risk	Some concerns	High risk
Nassib et al. (2022)	Low risk	Some concerns	Low risk	Some concerns	Some concerns	Some concerns
Sharma (2013)	Some concerns	Some concerns	Some concerns	High risk	Some concerns	High risk
Simonsmeier et al. (2018)	Low risk	Some concerns	Some concerns	Low risk	Some concerns	Some concerns
Veit et al. (2026)	Some concerns	Some concerns	Some concerns	Some concerns	Some concerns	Some concerns

Table 3 presents the risk of bias assessment for non-randomized intervention studies and observational designs appraised using ROBINS-I (framed around confounding, participant selection, exposure/intervention classification, deviations from intended interventions, missing data, outcome measurement, and selective reporting).

**Table 3:** Risk of bias in non-randomized studies.

Study ID	D1 confounding	D2 selection of participants	D3 classification of interventions/exposures	D4 deviations from intended interventions	D5 missing data	D6 measurement of outcomes	D7 selection of the reported result	Overall risk of bias
Ahmed et al. (2021)	Serious	Moderate	Low	Moderate	No information	Serious	Moderate	Serious
Calmels and Fournier (2001)	Serious	Low	Low	Moderate	Low	Low	Moderate	Serious
Calmels et al. (2006)	Serious	Moderate	Low	Moderate	Low	Low	Moderate	Serious
Di Rienzo et al. (2022)	Moderate	Moderate	Low	Low	Low	Low	Moderate	Moderate
Hökelmann et al. (2005)	Serious	Serious	Moderate	Moderate	Moderate	Serious	Serious	Serious
Napolitano (2017)	Critical	Serious	Moderate	Moderate	Moderate	Serious	Serious	Critical
Rymal and Ste-Marie (2017)	Serious	Moderate	Low	Moderate	Moderate	Low	Moderate	Serious
Simonsmeier and Buecker (2017)	Moderate	Moderate	Low	Low	Moderate	Low	Moderate	Moderate

### 3.4 Results of individual studies

Table 4 summarizes studies that evaluated imagery-based interventions or broader mental/psychological training programs implemented over multiple sessions and intended to improve gymnastics performance. Given substantial heterogeneity, interventions were additionally classified using a prespecified component taxonomy and summarized in a frequency table to make explicit which features are actually represented in the evidence base.

**Table 4:** Results of studies on imagery-based interventions and psychological training programs targeting performance.

Study	Within-subject effects of imagery training	Between-subjects comparing imagery with control	Main findings/conclusions
Ahmed et al. (2021)	Experimental group (*n* = 10) improved from pre to post on all targeted floor skills (10-point judging): side somersault on two hands with ¼ turn 2.80 ± 0.63 → 5.70 ± 1.25 (*t* = −6.539, *p* < 0.01); back flip on two hands 3.00 ± 0.67 → 6.20 ± 1.03 (*t* = −8.232, *p* < 0.01); back somersault straight 2.50 ± 0.53 → 5.50 ± 1.18 (*t* = −7.348, *p* < 0.01); total skill score 13.20 ± 1.62 → 29.00 ± 2.31 (*t* = −17.714, *p* < 0.01). Overall mental imagery score also increased (38.30 ± 3.92 → 53.00 ± 3.97; *t* = −8.333, *p* < 0.01; all subscales *p* < 0.01).	Post-intervention, the experimental group (*n* = 10) outperformed the control group (*n* = 10) on total floor skill score (31.60 ± 1.17 vs. 22.10 ± 1.10; *t* = 18.671, *p* < 0.01) and on each skill: side somersault with ¼ turn 6.40 ± 0.70 vs. 4.60 ± 0.52 (*t* = 6.548, *p* < 0.01); back flip 6.40 ± 0.70 vs. 4.40 ± 0.52 (*t* = 7.276, *p* < 0.01); back somersault straight 6.20 ± 0.63 vs. 4.30 ± 0.48 (*t* = 7.550, *p* < 0.01). Mental imagery was also higher in the experimental group (reported overall score 55.00 ± 3.97 vs. 38.30 ± 3.92; *t* = 9.466, *p* < 0.01).	An 8-week mental imagery program delivered alongside technical practice (3 sessions/week; 15 min/session; progressive relaxation, basic and multidimensional imagery) significantly improved beginners’ floor-skill execution and imagery ability compared with skill practice alone.
Battaglia et al. (2014)	Experimental group (*n* = 36) demonstrated significant pre–post improvements in jump reactivity/stiffness indices: HT flight time 0.476 ± 0.16 → 0.556 ± 0.12 (*Δ* = 16.8%; *p* < 0.01 vs. post), DJ flight time 0.501 ± 0.16 → 0.573 ± 0.15 (Δ = 14.4%; *p* < 0.01 vs. post), HT contact time 0.246 ± 0.15 → 0.192 ± 0.10 (Δ = −22.0%; *p* < 0.01 vs. post), and DJ contact time 0.201 ± 0.20 → 0.184 ± 0.21 (Δ = −8.6%; *p* < 0.01 vs. post). CMJ flight time changed minimally (0.568 ± 0.65 → 0.577 ± 0.65; Δ = 1.6%).	Repeated-measures MANOVA indicated significant effects of time (baseline vs. post), *F*(1,71) = 11.957, Wilk’s *Λ* = 0.695, *p* < 0.01; group (experimental vs. control), *F*(1,71) = 10.620, Wilk’s Λ = 0.719, *p* < 0.01; and time × group interaction, *F*(1,71) = 16.029, Wilk’s Λ = 0.629, *p* < 0.01. Bonferroni-adjusted comparisons showed superior post-training outcomes in the experimental group vs. control for HT flight time (0.556 ± 0.12 vs. 0.456 ± 0.14; *p* < 0.01), HT contact time (0.192 ± 0.10 vs. 0.254 ± 0.16; *p* < 0.01), and DJ flight time (0.573 ± 0.15 vs. 0.527 ± 0.15; *p* < 0.01). Reported between-group post-test effect sizes (Cohen’s *d*) were 0.08 (CMJ FT), 0.78 (HT FT), 0.22 (DJ FT), and 0.46 (HT CT).	A 6-week protocol combining unguided video observation (external model) with PETTLEP-based motor imagery immediately prior to physical jump practice improved key jumping-performance parameters in national-level rhythmic gymnasts relative to physical practice alone, with the largest effect for HT flight time (*d* = 0.78). Within the experimental group, imagery ability (MIQ-R) correlated with post-training HT flight time *r*(34) = 0.295 (*p* < 0.05) and DJ flight time *r*(34) = 0.297 (*p* < 0.05).
Guo (2014)	Reporting limitations: the study describes an imagery + conventional training group and a conventional-only control (*n* = 15 each; 16-week intervention), but Table 1 presents a single set of pre–post means ± SD with paired-test *p*-values (group attribution unclear). Reported pre–post changes were: Aerobic 8.766 ± 2.712 → 8.901 ± 2.956 (*p* = 0.04); Difficulty 7.466 ± 2.046 → 7.921 ± 2.236 (*p* = 0.05); Connection 8.133 ± 1.916 → 8.324 ± 2.063 (*p* = 0.05); Route 7.956 ± 2.126 → 8.023 ± 2.338 (*p* = 0.04); “Music with” 8.231 ± 2.512 → 8.648 ± 2.564 (*p* = 0.03); Total score 8.11 ± 1.884 → 8.426 ± 2.076 (*p* = 0.02).	No post-intervention between-group statistical comparisons (e.g., time × group) were reported. Baseline imagery (Movement Imagery Scale) did not differ between the experimental and control groups (all *p* > 0.05): Stimulate special motivation 25 ± 6 vs. 26 ± 5 (*t* = 0.84); Aroused and motivation 26 ± 4 vs. 25 ± 6 (*t* = 0.65); Excitation control motivation 28 ± 4 vs. 27 ± 4 (*t* = 0.94); Special recognition 24 ± 6 vs. 28 ± 3 (*t* = 1.14); General cognitive 22 ± 7 vs. 23 ± 7 (*t* = 1.08).	Across ~4 months, the authors conclude that integrating imagery practice with conventional aerobics training can improve routine performance (particularly route execution and music coordination) and enhance training engagement (greater self-reported liking of training and willingness to invest effort).
Hökelmann et al. (2005)	After 4 weeks of computer/video-assisted mental training (“Gymnastic mental”) implemented alongside practice, the experimental group showed a significant pre–post improvement in the cognitive component of movement representation (image-alignment/matching test) for the Kosak jump, Grand Jete, and a spread-leg jump with ½ turn, and improved expert-rated movement quality for these jumps.	No reported	This 4-week rhythmic-gymnastics study suggests that integrating structured mental training with concurrent motor practice can enhance both cognitive movement representation and expert-rated technique quality, with the greatest qualitative improvement reported for the coordinatively demanding spread-leg jump with ½ turn.
Khalaf et al. (2024)	Experimental group (*n* = 30) completed seven mental-training sessions (14:30 min each; relaxation, “controlling the controllables,” and video-supported mental imagery) delivered once per lesson over 7 weeks. Kinematic Coherence Scale scores (0–5 per stage) increased significantly from pre- to post-test across all six stages: Roundoff A 2.901 ± 0.328 → 4.628 ± 0.734 (*t* = 2.451, *p* < 0.001); B 1.315 ± 0.647 → 4.151 ± 0.477 (*t* = 2.383, *p* < 0.001); C 1.440 ± 0.347 → 4.381 ± 0.624 (*t* = 2.195, *p* = 0.004); Back handspring D 0.451 ± 0.834 → 4.208 ± 0.912 (*t* = 2.842, *p* < 0.001); E 0.507 ± 0.398 → 3.807 ± 0.124 (*t* = 2.173, *p* = 0.027); F 1.347 ± 0.664 → 4.981 ± 0.358 (*t* = 2.389, *p* < 0.001).	Post-test comparisons favored mental training (experimental *n* = 30) versus conventional instruction (control *n* = 32) for every performance stage: Roundoff A 4.628 ± 0.734 vs. 3.102 ± 0.711 (*t* = 11.560, *p* < 0.001; effect size = 0.143); B 4.151 ± 0.477 vs. 2.721 ± 0.354 (*t* = 18.157, *p* = 0.001; effect size = 0.149); C 4.381 ± 0.624 vs. 3.217 ± 0.787 (*t* = 8.905, *p* < 0.001; effect size = 0.107); Back handspring D 4.208 ± 0.912 vs. 2.872 ± 0.234 (*t* = 10.950, *p* < 0.001; effect size = 0.121); E 3.807 ± 0.124 vs. 3.201 ± 0.721 (*t* = 6.365, *p* < 0.001; effect size = 0.102); *F* 4.981 ± 0.358 vs. 3.921 ± 0.924 (*t* = 4.701, *p* < 0.001; effect size = 0.137).	Seven brief mental-training sessions embedded within instructional lessons produced larger improvements in expert-rated kinematic coherence for the roundoff–back handspring sequence than conventional feedback alone, with statistically higher post-test scores across all six movement stages.
Marshall and Gibson (2017)	Intervention group (*n* = 11) increased CSAI-2 self-confidence from pre- to post-test (21.09 ± 4.30 → 26.18 ± 4.38; marked significant by authors). Cognitive anxiety decreased (19.55 ± 4.59 → 17.82 ± 4.66) and somatic anxiety decreased (20.64 ± 6.86 → 18.55 ± 6.62). On the SIQ, MG-A increased (3.17 ± 1.25 → 4.02 ± 1.02; *F*1,17 = 7.16, p = 0.02, η^2^ = 0.30) and MG-M increased (4.11 ± 0.91 → 4.91 ± 1.27; F1,17 = 5.16, *p* = 0.04, η^2^ = 0.23).	Compared with control (*n* = 8), there was no significant group × time interaction for cognitive anxiety (*F*1,17 = 1.96, *p* > 0.05, η^2^ = 0.10), somatic anxiety (F1,17 = 0.92, *p* > 0.05, η^2^ = 0.35), any SIQ subscale (all *p* > 0.05), or acrobatic performance score (F1,17 = 0.82, *p* = 0.38, η^2^ = 0.13). Self-confidence increased significantly more in the imagery group (group × time: *F*1,17 = 14.18, *p* = 0.002; reported effect size *d* = 0.46). Baseline performance differed between groups (independent t-test *p* < 0.05), with higher pre-test performance in the intervention group.	A 4-week imagery-script intervention (2 sessions/week; ~15 min/session) improved pre-competitive self-confidence in national-level acrobatic gymnasts, but did not significantly reduce cognitive or somatic anxiety and did not improve competition performance.
Napolitano (2017)	Uncontrolled two-athlete case study (female artistic gymnasts aged 12–15 y; ≥5 years experience) using motor imagery (first- and third-person) over 5 months. Judge scores for the round-off flic increased across 11 assessments for both athletes: Athlete 1 from 6.3 (assessment 1) to 8.5 (assessment 11; Δ = +2.2 points), and Athlete 2 from 6.5 to 8.5 (Δ = +2.0 points). Athletes’ hetero-evaluations also increased over time (Athlete 1 rating Athlete 2: 3.5 → 8.2; Athlete 2 rating Athlete 1: 3.0 → 9.0), with marked improvement observed in the later months.	No reported	Across 5 months of combined first-person and third-person motor imagery practice supported by self- and hetero-evaluation (and video-based prediction in the second study phase), the authors report performance improvement and improved evaluative consistency, with ~80% of ratings showing improvement over time. Athletes’ early hetero-evaluations did not align with the judge’s scores (tending to underestimate performance), but convergence/consistency improved in later months.
Rymal and Ste-Marie (2017)	Across four uneven-bars competitions, execution scores (0–10) for the FF-VSM + ATP group (*n* = 10) were: early season with video (EV) 8.70 ± 0.79 and without video (ENV) 8.87 ± 0.39; late season with video (LV) 9.29 ± 0.20 and without video (LNV) 8.86 ± 0.89. For the FF-VSM-only group (*n* = 8), scores were: EV 8.59 ± 0.68; ENV 8.69 ± 0.47; LV 9.19 ± 0.29; LNV 8.87 ± 0.32. Both groups showed higher mean execution scores late vs. early in the season (overall early *M* = 8.72, SD = 0.45; late *M* = 9.05, SD = 0.58).	Mixed 2 (group: FF-VSM + ATP vs. FF-VSM-only) × 2 (condition: video vs. no-video) × 2 (time: early vs. late) ANOVA (repeated measures on condition and time) showed a main effect of time, *F*(1,16) = 14.76, *p* = 0.001, η^2^ = 0.48, with higher execution scores late vs. early season; no main effects of group or condition and no interactions were observed (all *F*s < 1.0). When visual imagery ability (MIQ-R visual; median split) was tested as a moderator, the time effect was superseded by a visual-imagery group × time × condition interaction, *F*(1,16) = 5.976, *p* = 0.026, η^2^ = 0.27: gymnasts high in visual imagery showed no statistically significant benefit of FF-VSM at early or late competitions, whereas those low in visual imagery exhibited a decrement when FF-VSM was used early in the season and an advantage when FF-VSM was used late in the season.	In this competition-embedded feedforward video self-modeling (FF-VSM) study, a 4-week advanced training program (attention focus, imagery, goal-setting, and self-instruction) did not enhance the effects of FF-VSM on uneven-bars execution scores, and FF-VSM itself showed no overall performance benefit versus no-video competitions. Performance improved across the season irrespective of condition. Exploratory moderation analyses suggested FF-VSM may be more beneficial for gymnasts with lower visual imagery ability (MIQ-R), particularly later in the competitive season. Interview data indicated broadly similar self-regulatory processes reported by both groups when using FF-VSM.
Sharma (2013)	Pre- and post-intervention floor exercise performance was assessed using FIG Code of Points (2009–2012), but the article does not report pre-test and post-test means separately or paired pre–post test statistics. Post-test descriptive performance scores were reported by group and sex: PST group *n* = 28, *M* = 9.23, SD = 2.69 (girls *n* = 12: 10.05 ± 1.36; boys *n* = 16: 8.62 ± 3.29) and control group *n* = 24, *M* = 6.65, SD = 3.55 (girls *n* = 12: 6.44 ± 3.03; boys *n* = 12: 6.87 ± 4.13).	ANCOVA (DV: post-test floor score; covariate: pre-test score) showed a significant adjusted group effect favoring PST vs. control, *F*(1,47) = 4.51, *p* = 0.039 (model *R*^2^ = 0.772; adjusted *R*^2^ = 0.753). The adjusted mean difference was 1.039 (SE = 0.489), 95% CI [0.056, 2.022] (LSD), indicating higher post-test performance in the PST group after controlling for baseline performance. Neither sex [*F*(1,47) = 2.47, *p* = 0.12] nor the group × sex interaction [*F*(1,47) = 0.428, *p* = 0.51] was statistically significant.	In Indian artistic gymnasts aged 9–17 years (completers: PST *n* = 28; control *n* = 24), a 6-week psychological skills training program (30–45 min/session, 5 days/week; goal-setting, relaxation, self-talk, imagery, attention, and confidence) delivered in addition to regular physical training was associated with higher judged floor exercise performance at post-test compared with regular training alone after adjustment for baseline performance; effects did not differ by sex.
Simonsmeier et al. (2018)	Field-based 4-week imagery phase (audio-guided script; 4 sessions/week; 3 imagery repetitions/session; 48 total imagined repetitions) was embedded within regular practice in a cross-over design (imagery-first vs. imagery-last). Performance (cast to handstand on bars) was indexed as a total error score (0–7; lower = better). During the imagery phase, high-expertise gymnasts in the imagery-last sequence (*n* = 13) showed a significant reduction in error score from T2 to T3 (3.02 ± 1.35 → 2.39 ± 0.84; *p* = 0.04; *d* = 0.88). High-expertise gymnasts in the imagery-first sequence (*n* = 9) improved from T1 to T2 (2.98 ± 0.77 → 2.43 ± 0.95), but this did not reach statistical significance (*p* = 0.09; *d* = 0.56). Low-expertise gymnasts did not improve during their imagery phase (imagery-first: 3.92 ± 1.24 → 3.92 ± 1.28; imagery-last: 3.63 ± 0.85 → 3.62 ± 1.04; all *p*s ≥ 0.48). Mental representation (SDA-M) changed during the imagery phase in the total imagery-last group (*λ* = 0.46 for T2 vs. T3) with increased similarity to the expert reference cluster solution (ARI 0.03 → 0.31), whereas no representational change was observed for the total imagery-first group during its imagery phase (λ = 0.70 for T1 vs. T2).	Repeated-measures ANOVA (time [T1, T2, T3] × sequence [imagery-first vs. imagery-last] × expertise [low vs. high]) on error scores showed a main effect of expertise (*F* = 13.05, *p* < 0.001; ηp^2^ = 0.25), indicating consistently lower error scores in high- versus low-expertise gymnasts across measurement points. There was also a significant time × sequence × expertise interaction (*F* = 5.59, *p* = 0.01; ηp^2^ = 0.13). No main effects of time (*F* = 2.53, p = 0.09; ηp^2^ = 0.06) or sequence/group (*F* = 0.04, *p* = 0.84; ηp^2^ = 0.00) were observed, and the time × sequence and time × expertise interactions were not significant (*F*s ≤ 1.98, *p*s ≥ 0.15). During the regular-training (control) phase, no subgroup showed significant performance change (all *p*s ≥ 0.11).	In 56 female gymnasts aged 7–15 years, a short, practice-integrated imagery program improved cast-to-handstand performance only under specific conditions: statistically significant gains were confined to high-expertise gymnasts who completed imagery after an initial period of regular practice (imagery-last; *d* = 0.88), whereas low-expertise gymnasts showed no detectable benefit. Changes in mental movement representations (SDA-M) were heterogeneous across groups, yielding an overall pattern consistent with performance benefits that are moderated by expertise level and intervention sequencing.

Table 5 summarizes studies that did not primarily test a multi-session imagery training program for performance enhancement, but instead examined imagery-related mechanisms and processes, including mental chronometry, postural/psychophysiological responses during imagery, acute comparisons of mental simulation strategies, associations between imagery variables and performance, and the effects of attentional instruction. These studies contribute complementary evidence on how imagery may operate in gymnasts and which contextual or athlete-level factors may shape responsiveness.

**Table 5:** Results of individual studies focusing on imagery processes, acute effects, and performance associations.

Study	Within-subject effects of imagery training	Between-subjects comparing imagery with control	Main findings/conclusions
Calmels and Fournier (2001)	In 12 elite female artistic gymnasts (age 13–20 years; *M* = 16, SD = 2), routine duration was compared across physical execution vs. mental execution (imagery) over three trials. A 7 (trial: 1–3) × 2 (condition) repeated-measures ANOVA showed no trial effect (*p* < 0.0071) and no trial × condition interaction (*p* < 0.0071), but a significant main effect of condition for the entire routine and five of six routine stages: entire routine 83.14 s (physical) vs. 65.33 s (mental), *F*(1,11) = 55.62, *p* < 0.0001, ES = 1.91; Stage 1 9.39 vs. 7.77 s, *F*(1,11) = 12.58, *p* < 0.0046, ES = 0.49; Stage 2 11.62 vs. 9.88 s, *F*(1,11) = 16.93, *p* < 0.0017, ES = 0.51; Stage 3 15.99 vs. 10.61 s, *F*(1,11) = 24.74, *p* < 0.0004, ES = 1.23; Stage 5 18.49 vs. 14.10 s, *F*(1,11) = 25.20, *p* < 0.0004, ES = 0.74; Stage 6 14.07 vs. 11.19 s, *F*(1,11) = 11.98, *p* < 0.0053, ES = 0.78. Stage 4 showed a non-significant trend after Bonferroni correction (13.56 vs. 11.78 s, *F*(1,11) = 8.94, *p* < 0.0123, ES = 0.46). Relative stage durations (stage time/total time) were broadly similar across conditions; the largest directional differences were Stage 3 (physical 0.190 vs. mental 0.161; ≈15% longer under physical execution) and Stages 2 and 4 (mental 0.151 vs. physical 0.138; mental 0.185 vs. physical 0.167; each ≈10% longer under imagery).	Not applicable	Across three repeated trials, elite artistic gymnasts mentally executed (imagery) a floor routine faster than they physically performed it, with statistically robust reductions in total duration and in most routine stages. The temporal structure (relative stage contributions) was largely preserved, although stage-specific shifts suggested that perceived element difficulty may differentially compress or expand imagery timing.
Calmels et al. (2006)	In 16 elite female artistic gymnasts (age 12–18 years; M = 14.5, SD = 1.63), chronometric data were collected for a Yurchenko vault performed under imagined vs. actual conditions across three trials, with the vault segmented into four phases (Stage 1: run; Stage 2: first flight; Stage 3: arm support; Stage 4: second flight). For the full vault, a 2 (perspective: first- vs. third-person) × 3 (trial) × 2 (condition: imagined vs. actual) mixed ANOVA found no main effects or interactions, including no condition effect, *F* = 2.631, *p* = 0.13. Descriptively, averaged across trials, first-person imagery vs. actual times were 4.91 ± 1.95 s vs. 5.84 ± 0.40 s; third-person imagery vs. actual times were 5.65 ± 1.49 s vs. 6.29 ± 0.40 s. For stage times, the 2 × 4 (stage) × 3 (trial) × 2 (condition) ANOVA showed a significant stage × condition interaction, *F*(3,42) = 125.9179, *p* < 0.000001, indicating stage-specific temporal distortion under imagery: Stage 1 was significantly faster in imagery than in actual execution (*p* < 0.001), whereas Stages 2 and 3 were significantly slower in imagery than in actual execution (Stage 2 *p* < 0.0004; Stage 3 *p* < 0.0003); Stage 4 did not differ between conditions. Stage means (averaged across trials) illustrated this pattern in first-person imagery vs. actual: Stage 1 2.18 ± 0.75 vs. 4.48 ± 0.41 s; Stage 2 0.90 ± 0.49 vs. 0.31 ± 0.03 s; Stage 3 0.77 ± 0.54 vs. 0.20 ± 0.04 s; Stage 4 1.05 ± 0.49 vs. 0.85 ± 0.08 s; and in third-person imagery vs. actual: Stage 1 2.69 ± 1.05 vs. 4.92 ± 0.38 s; Stage 2 0.98 ± 0.42 vs. 0.33 ± 0.03 s; Stage 3 0.87 ± 0.45 vs. 0.20 ± 0.02 s; Stage 4 1.11 ± 0.45 vs. 0.83 ± 0.09 s. A stage × trial interaction was also reported, *F*(6,84) = 2.7672, *p* < 0.02, reflecting a small increase in Stage-1 duration from Trial 1 (3.504 s) to Trial 3 (3.621 s) irrespective of perspective and condition.	No between-group intervention comparison was conducted; the between-subject factor was imagery perspective (first-person vs. third-person). Perspective did not significantly affect full-vault duration (main effect *F* = 1.475, *p* = 0.24) and did not interact with condition (perspective × condition *F* = 0.092, *p* = 0.77) or with trial (perspective × trial *F* = 1.960, *p* = 0.16) for the full-vault analysis. For stage analyses, there was no main effect of perspective (*F* = 1.475, *p* = 0.24) and no evidence that perspective modified the imagery–actual discrepancy (perspective × stage × condition *F* = 0.004, *p* = 0.99; perspective × condition *F* = 0.092, *p* = 0.77). The perspective × stage interaction approached but did not reach conventional significance (*F* = 2.512, *p* = 0.07).	Elite gymnasts showed comparable total durations when imagining vs. physically performing a complex vault (no overall condition effect), regardless of whether imagery was generated from a first-person or third-person perspective. However, stage-level analyses demonstrated clear temporal reorganization under imagery, since the run-up phase was compressed, whereas the two brief aerial/support phases were expanded, with the landing phase largely preserved. Thus, temporal functional equivalence was supported only for the full-vault aggregate, not for the vault’s component phases, and imagery perspective did not meaningfully moderate these effects.
Di Rienzo et al. (2022)	Cross-sectional repeated-measures (no imagery training). Across mental-practice conditions (visual MI, kinesthetic MI, and mental-calculation control), stabilometric indices differed by condition for COP surface [*F*(2,52) = 10.65, *p* < 0.001, ηp^2^ = 0.29], ML-length [*F*(2,52) = 5.05, *p* = 0.009, ηp^2^ = 0.16], and length-by-surface (F(2,52) = 4.31, p = 0.02, ηp^2^ = 0.14). *Post hoc* contrasts showed greater COP surface during KMI vs. CONTROL [p(*k* = 3) < 0.001] and vs. VMI [*p*(k = 3) = 0.01], with no difference between VMI and CONTROL [*p*(k = 3) = 0.13]. ML-length was greater during KMI vs. VMI [*p*(*k* = 3) = 0.007]. In planned contrasts, EXPERTS (but not NON-EXPERTS) showed reduced length-by-surface during VMI and KMI compared with CONTROL [both *p*(*k* = 3) = 0.04].	Experts (*n* = 13) vs. non-experts (*n* = 15): baseline stance COP variables did not differ between groups (all *p* > 0.05). During physical execution of the arabesque (actual practice), experts exhibited reduced AP-length relative to non-experts (646.37 ± 169.26 vs. 692.06 ± 188.46 mm; *F*(1,26) = 3.23, *p* = 0.04, ηp^2^ = 0.11), with no group differences for COP surface (2,451.63 ± 1,118.21 vs. 2,316.05 ± 913.41 mm^2^; *F*(1,26) = 0.43, *p* = 0.51), ML-length [624.05 ± 143.79 vs. 656.33 ± 136.36 mm; *F*(1,26) = 0.56, *p* = 0.46], or length-by-surface [0.48 ± 0.29 vs. 0.51 ± 0.32 mm^−1^; *F*(1,26) = 0.11, *p* = 0.73]. For mental-practice conditions, the CONDITION × GROUP interaction affected AP-length [*F*(2,52) = 2.38, *p* = 0.04, ηp^2^ = 0.09] and was borderline for length-by-surface [*F*(2,52) = 3.03, *p* = 0.05, ηp^2^ = 0.10]; difference-of-differences indicated that the CONTROL→VMI and CONTROL→KMI decreases in length-by-surface observed in experts were absent in non-experts [both p(*k* = 3) = 0.04], while analogous AP-length slope differences were marginal [both p(*k* = 3) = 0.09]. Subjective judge ratings of arabesque performance did not differ by group [*F*(1,26) = 0.22, *p* = 0.64]. Visual MI vividness ratings were higher in experts (4.96 ± 0.85) than non-experts [3.60 ± 0.66; *F*(1,26) = 14.53, *p* < 0.001, ηp^2^ = 0.35], whereas KMI vividness did not differ [*F*(1,26) = 1.05, *p* = 0.30]. On the VMIQ-2, non-experts reported greater difficulty for external visual imagery (36.93 ± 10.03) than experts [26.62 ± 5.66; *F*(1,26) = 10.71, *p* = 0.003, ηp^2^ = 0.28].	In this stabilometric experiment, gymnastics expertise was associated with lower antero-posterior COP displacement during a one-leg balance skill and with distinct postural modulation during imagery: experts reduced AP-length and length-by-surface during both visual and kinesthetic MI relative to a mental-calculation control, whereas non-experts showed minimal modulation across conditions. Experts also demonstrated superior visual imagery (higher VMI vividness and better VMIQ-2 external visual imagery scores). Stabilometric correlates of VMI predicted corresponding indices during actual practice [Surface: *F*(1,23) = 5.50, *p* = 0.01, ηp^2^ = 0.19; ML-length: *F*(1,23) = 3.99, *p* = 0.02, ηp^2^ = 0.15; AP-length: *F*(1,23) = 3.24, *p* = 0.04, ηp^2^ = 0.12], supporting a functional link between visual imagery-related postural activity and physical execution of the balance skill.
Nassib et al. (2022)	Immediate effects were assessed in 18 male gymnasts performing a standing jump backward tucked (technical score /20), using two experimental sessions (mental imagery; video-based visualization) with baseline performance measured before each strategy. ANCOVA (repeated-measures ANOVA with baseline performance as covariate) indicated a significant effect for performance “with mental simulation” (*F* = 5.312, *p* = 0.05, ηp^2^ = 0.431), whereas baseline performance “without mental simulation” was not significant (*F* = 3.959, *p* = 0.087, ηp^2^ = 0.361); a main effect of training was also observed (*F* = 12.556, *p* = 0.001, ηp^2^ = 0.611). The interaction between imagery and visualization was significant (*F* = 8.46, *p* ≤ 0.05; Bonferroni *post hoc p* < 0.05). Post-strategy performance means were IM 13.277 ± 1.655 (*n* = 9) and visualization 12.722 ± 1.986 (*n* = 9), corresponding to a 6.14% higher score for IM relative to visualization.	Not applicable	Across the two acute mental-simulation strategies, performance improved relative to baseline and differed by strategy, with mental imagery producing a modestly higher mean performance score than visualization (≈ + 6.1%). Psychologically, the authors report improved self-confidence and self-evaluation after mental strategies, with self-evaluation positively correlated with self-confidence both after training without mental strategies (*r* = 0.889, *p* ≤ 0.001) and after visualization (*r* = 0.680, *p* ≤ 0.05). Self-confidence was negatively correlated with performance without mental stimulation (*r* = −0.709, *p* ≤ 0.05) and with performance after visualization (*r* = −0.759, *p* ≤ 0.05).
Simonsmeier and Buecker (2017)	Cross-sectional observational study in 75 competitive gymnasts (7–16 y; *M* = 10.87 ± 2.76) examining whether imagery use (SIQ-C) and imagery ability (SIAQ; 5-point scale) predict competition performance (mean performance percentage 81.80% ± 0.12; range 46–95%). Five separate multiple regressions (matched SIQ-C subscales with corresponding SIAQ subscales) showed significant unique predictors of performance for: cognitive specific imagery use [*β* = 0.24, *t*(73) = 2.03, *p* = 0.05; model *R*^2^ = 0.08], motivational general–mastery imagery use [*β* = 0.30, *t*(73) = 2.36, *p* = 0.02; model *R*^2^ = 0.33], and motivational specific (goal) imagery ability [*β* = 0.30, *t*(73) = 2.50, *p* = 0.02; model *R*^2^ = 0.17]. Imagery ability did not moderate the imagery use–performance relationship in any model (all use × ability *p* > 0.14); controlling for age did not change results.	Not applicable	In young competitive gymnasts, higher reported use of cognitive specific and motivational general–mastery imagery, and greater ability to image goals, were associated with better competition performance, independent of age. Imagery use and imagery ability were positively interrelated (CS–Skill *r* = 0.23, *p* < 0.05; MS–Goal *r* = 0.35, *p* < 0.01; MG-M–Mastery *r* = 0.31, *p* < 0.01), but imagery ability did not moderate the imagery use–performance association. Post hoc analyses suggested higher imagery (cognitive specific use and goal imagery ability) related to fewer execution deductions (negative correlations with *E*-score), with no association with difficulty (*D*-score).
Veit et al. (2026)	Across all participants (*N* = 29) completing a baseline block (6 trials) and four instruction phases (4 × 5 trials), descriptive performance metrics showed a progressive deterioration from baseline to Phase 4 (see Supplementary Table 2): deviation from the targeted 450° longitudinal-axis turn (LAD) increased in magnitude from −27.10 ± 29.16° at baseline to −65.15 ± 36.12° at Phase 4; jump height decreased from 22.73 ± 3.27 to 21.40 ± 3.55 cm; forward/reverse body alignment at 270° (Body1) increased from 3.26 ± 2.23° to 6.14 ± 5.04°; sideways body alignment at 360° (Body2) increased from 3.70 ± 2.62° to 5.86 ± 4.80°; and steps after landing increased from 0.65 ± 0.56 to 1.33 ± 0.94. Mixed-effects modeling confirmed significant phase effects for LAD [χ^2^(4) = 75.079, *p* < 0.001], Height [χ^2^(4) = 19.104, *p* < 0.001], Hip [χ^2^(4) = 6.448, *p* < 0.001], Body1 [χ^2^(4) = 14.756, *p* < 0.001], and Body2 [χ^2^(4) = 6.667, *p* < 0.001], but not Knee [*χ*^2^(4) = 2.058, *p* = 0.085].	Instruction groups (match *n* = 9; no-match *n* = 11; neutral *n* = 9) did not differ in age, expertise, or imagery vividness [age: *F*(2,26) = 0.823, *p* = 0.450, η^2^ = 0.060; expertise: *F*(2,26) = 1.352, *p* = 0.276, η^2^ = 0.094; vividness: *F*(2,26) = 3.075, *p* = 0.063, η^2^ = 0.191]. Linear mixed-model likelihood-ratio tests showed no instruction-group effects for LAD [χ^2^(2) = 1.735, *p* = 0.200], Height [χ^2^(2) = 0.012, *p* = 0.913], Hip [χ^2^(2) = 0.001, *p* = 0.974], Knee [χ^2^(2) = 0.061, *p* = 0.806], Body1 [χ^2^(2) = 1.570, *p* = 0.222], or Body2 [χ^2^(2) = 0.755, *p* = 0.394]. Similarly, logistic models indicated no group differences for Legs1 [χ^2^(2) = 0.260, *p* = 0.771], Legs2 [χ^2^(2) = 1.175, *p* = 0.309], or Steps [χ^2^(2) = 1.093, *p* = 0.336].	In women’s artistic gymnasts (Mage = 19.55 ± 3.01 years; expertise = 11.38 ± 4.24 years), externally focused, internally focused, and neutral verbal instructions produced no detectable between-group differences in stretched jump performance with a 450° longitudinal-axis turn, including when instructions were classified as matching versus not matching athletes’ most vivid imagery type (VMIQ-2). Performance declined across successive instruction phases (significant phase effects for five of nine variables), consistent with a disruptive effect of consciously directing attention and/or fatigue during repeated trials, rather than a beneficial effect of a specific instruction focus.

## 4 Discussion

The evidence synthesized in this review suggests that imagery-based and broader psychological skills interventions may be associated with improvements in selected gymnastics-related performance outcomes, but confidence in a causal improvement claim is limited given the predominance of studies rated as some concerns/high risk (RoB 2) and serious/critical (ROBINS-I), alongside substantial heterogeneity in outcomes and intervention content. Across controlled and quasi-controlled designs, several studies reported meaningful benefits in judged skill execution or task performance following structured imagery or multi-component psychological skills training. In contrast, other trials found null effects on performance despite improvements in psychological variables such as self-confidence, while acute mental simulation strategies produced modest short-term differences that did not map cleanly onto stable, competition-relevant performance changes. The results support imagery as a potentially useful adjunct to physical practice, but they also stress that expertise level, intervention dose and sequencing, outcome selection, and methodological quality likely moderate observed effects.

### 4.1 Performance outcomes in gymnastics tasks and judged routines

Across studies directly assessing gymnastics performance (judged execution, skill scores, or task-specific error metrics), the most consistent pattern was that imagery integrated alongside training can coincide with improved performance, particularly when imagery is structured and tightly coupled to motor execution demands. Substantial pre–post improvements in specific floor-skill scores were reported following an imagery program delivered in parallel with technical practice in beginners (Ahmed et al., 2021). However, because this evidence comes from a quasi-experimental pre–post design, apparent benefits may be inflated by baseline imbalance, and outcome-measurement limitations. Similarly, in youth artistic gymnasts, a multi-component psychological skills training package that included imagery was associated with higher post-test floor exercise scores than standard training after adjustment for baseline performance (Sharma, 2013). In an applied instructional context, brief mental-training sessions incorporating relaxation and video-supported imagery improved expert-rated kinematic coherence of a roundoff–back handspring sequence relative to conventional instruction (Khalaf et al., 2024). These findings align with a motor-learning account in which imagery refines action representation via functional equivalence between simulated and executed actions, updating of predictive internal models used for feedforward control and error-based correction, and common-coding/event-file principles whereby perceptual and motor features are bound into shared sensorimotor representations that can be reactivated by imagery (Jeannerod, 1995).

However, positive effects were not universal. A controlled imagery-script intervention improved self-confidence but did not improve acrobatic competition performance (Marshall and Gibson, 2017), critically, the authors reported a significant baseline between-group difference in acrobatic performance, and the primary analysis for performance relied on the group × time test without a baseline-adjusted model, limiting inference about null effects. Evidence from a cross-over design further indicates that performance benefits may be conditional, since imagery improved cast-to-handstand errors only in higher-expertise gymnasts and only when imagery occurred after an initial period of regular practice (Simonsmeier et al., 2018). This is consistent with the notion that motor imagery can support the development or refinement of motor schemas and improve the organization and retrieval of movement plans under competitive pressure. However, alternative accounts are also plausible in gymnastics, including attentional control/reinvestment models in which imagery changes the locus/robustness of attentional control and susceptibility to conscious movement monitoring under pressure, and constraint-led/ecological dynamics perspectives in which imagery may function by stabilizing perception–action couplings and task-relevant information pickup rather than by strengthening a stored ‘schema’ per se. In gymnastics, these accounts make partially discriminable predictions, thus schema/internal-model accounts predict larger benefits on structurally similar skills and on representation-sensitive measures, whereas reinvestment accounts predict that imagery benefits should be moderated by trait/state reinvestment and should be accompanied by reduced breakdowns under pressure even when baseline technical proficiency is matched.

A subset of included studies reported improvements without fully supporting causal inference because of limited reporting or design constraints. For example, performance improvements over time were described in aerobics athletes participating in imagery plus conventional training, but group-specific outcome reporting and between-group analyses were insufficient to quantify imagery-specific effects (Guo, 2014). Other study also described improvements attributed to mental training but did not provide adequate statistical detail for robust interpretation (Hökelmann et al., 2005).

### 4.2 Physical and biomechanical proxies relevant to performance

Some studies classified performance through physical or biomechanical proxies that are plausibly relevant to gymnastics execution and landings. In rhythmic gymnasts, a protocol that combined video observation with PETTLEP-oriented imagery, delivered immediately prior to physical practice, improved jump-related measures (e.g., flight time and contact time) and outperformed physical practice alone on several post-test outcomes (Battaglia et al., 2014). Although these outcomes are not identical to judged routine scores, they index reactivity and stiffness qualities that can influence execution quality, amplitude, and landing control. However, in synthesis they were treated as lower-evidential-weight outcomes because surrogate biomechanical measures (and expert-rated proxies) are more vulnerable to outcome-measurement bias and construct-mismatch than official competition endpoints, and therefore were down-weighted relative to judged routine/skill performance when drawing overall conclusions.

That said, the proxy-heavy outcome mix creates a clear ecological validity problem, since improvements on component-level proxies are necessary for understanding mechanism and skill elements, but are not sufficient for inferring meaningful change in routine-level, competition-scored performance unless representativeness and transfer are demonstrated. This gap is evident where psychological improvements occur without performance change (Marshall and Gibson, 2017) and reinforces the need for outcomes that are both sensitive and competition-relevant. Accordingly, we interpret the evidence using a multi-level performance model in which changes in isolated components (e.g., jump flight/contact parameters or kinematic coordination indices) may influence element execution quality and landing deductions, which likely aggregates into routine-level execution and, together with difficulty composition and competition constraints (pressure, fatigue, judging variability), determines the final competition outcome.

### 4.3 Psychological and self-regulatory outcomes

Several studies suggest imagery and related psychological interventions can influence self-regulatory variables, which are theoretically important for performance stability in judged sports. Improved self-confidence following imagery scripting (Marshall and Gibson, 2017) is particularly salient for gymnastics, where confidence can influence commitment to skills, initiation timing, and willingness to execute difficult elements. Acute mental simulation strategies were also associated with self-evaluation and self-confidence patterns that correlated with performance indices, albeit sometimes in counterintuitive directions (Nassib et al., 2022). Such findings may reflect complex situational dynamics. For example, heightened confidence might co-occur with riskier execution strategies, or athletes may recalibrate self-evaluations as they become more aware of technical deficits during focused mental practice.

Evidence also points to potential interactions between imagery ability and training response. A study (Battaglia et al., 2014) reported associations between imagery ability and post-training jump performance indices, implying that individual differences in imagery vividness/control may condition responsiveness to imagery-based protocols. Possibly, imagery is not a uniform dose, but a cognitive-motor skill that varies by athlete and can constrain intervention effectiveness unless explicitly trained and monitored.

### 4.4 Moderators of effect: expertise, sequencing, and instruction matching

Across studies, expertise emerged as a plausible moderator. In the cross-over imagery trial, only higher-expertise gymnasts improved during their imagery phase, and the magnitude and significance of change depended on whether imagery was delivered first versus last (Simonsmeier et al., 2018). This suggests that imagery may work best when athletes already possess stable coordinative structures and when imagery is introduced after foundational practice has established a reliable movement reference. Sequencing may matter because early imagery can amplify error representations or inconsistent movement patterns if physical execution is not yet stabilized; later imagery may instead consolidate a more accurate internal model.

Relatedly, evidence that matching instructions to athletes’ imagery preferences improves outcomes was not supported in the included instruction-focus study. Manipulating instruction focus (external, internal, neutral) and classifying instructions as matching versus not matching athletes’ imagery vividness profiles did not yield group differences; instead, performance declined across repeated phases (Veit et al., 2026). While this study is not a traditional imagery-training intervention, it raises an important caution for applied practice, since adding conscious attentional directives or cognitively demanding cues can be counterproductive, potentially through reinvestment processes or simple accumulated fatigue. In practical terms, imagery interventions may need to prioritize automaticity-supportive content and avoid overloading athletes with prescriptive attentional control during execution.

### 4.5 Studies limitations

Across the intervention studies, the most recurrent threats to internal validity were weak or poorly reported randomization/allocation procedures, non-blinding with subjectively judged outcomes, and incomplete reporting of attrition and analysis sets. Several parallel-group trials stated that participants were “randomly” allocated but did not provide adequate details to judge sequence generation or allocation (Guo, 2014; Marshall and Gibson, 2017; Sharma, 2013), while a study (Khalaf et al., 2024) described an even-number draw procedure that appears potentially predictable, raising concern for systematic baseline differences. Performance outcomes were frequently judge-scored without explicit blinding of judges to group or timepoint (Ahmed et al., 2021; Guo, 2014; Khalaf et al., 2024; Marshall and Gibson, 2017; Sharma, 2013), which is especially consequential in gymnastics where evaluator expectations can influence execution/artistry ratings. Relatedly, differential attrition or exclusions were not consistently addressed. For example, a study (Sharma, 2013) reported unequal completion between groups, and some trials provided limited clarity regarding missing outcome handling or per-protocol exclusions (Ahmed et al., 2021; Guo, 2014; Marshall and Gibson, 2017). Even in better-controlled designs with objective instrumentation or blinded video scoring (Battaglia et al., 2014; Simonsmeier et al., 2018), selective reporting risk remained difficult to dismiss because protocols/registrations were generally not referenced, and complex multi-outcome designs or phase-based protocols increase analytic degrees of freedom (Nassib et al., 2022; Veit et al., 2026).

Methodological limitations were more pronounced in the non-randomized and observational evidence base, where confounding and design-related biases were pervasive. Several studies used within-subject comparisons with fixed order (imagery before physical execution), which makes time, warm-up, fatigue, or learning effects plausible alternative explanations (Calmels et al., 2006; Calmels and Fournier, 2001). Other non-randomized intervention studies included non-equivalent groups defined by age/level or self-selection, along with incomplete counterbalancing across conditions, leaving residual confounding likely even when outcomes were based on official judges’ scores (Rymal and Ste-Marie, 2017). Observational cross-sectional designs comparing experts and non-experts or relating imagery measures to competition performance were also vulnerable to confounding by training volume, competitive level, and age when adjustment was limited or absent (Di Rienzo et al., 2022; Simonsmeier and Buecker, 2017). The uncontrolled case report design in a study (Napolitano, 2017) is intrinsically highly susceptible to maturation/training-cycle effects and selective reporting. These patterns suggest that future studies should prioritize prospective registration with prespecified primary outcomes aligned to discipline-style judged performance endpoints (e.g., routine/element execution scores) and analysis plans, and should adopt cluster randomization by club/coach (to reduce contamination of coaching cues and training context) when individual randomization is impractical. Where judged outcomes are used, performance should be scored by blinded external judges (or blinded video-based panels) using standardized criteria, with assessor blinding and scoring procedures reported explicitly, because lack of blinding is a recurrent driver of bias in this evidence base.

Imagery competence (e.g., vividness, controllability, and task-specific representational fidelity) is plausibly trainable and should be treated as an intermediate outcome within imagery interventions, not only as a baseline moderator measured once. Accordingly, future gymnastics trials should measure imagery competence longitudinally (pre–mid–post) and incorporate fidelity checks so that null performance effects can be interpreted against whether imagery capacity actually changed and whether the intended imagery dose/quality was achieved.

### 4.6 Practical considerations

The interventions that showed clearer performance benefits tended to be structured and integrated with training, rather than delivered as a generic add-on. Protocols incorporating structured scripts (Marshall and Gibson, 2017), PETTLEP-relevant elements and timing immediately prior to execution (Battaglia et al., 2014), or repeated, session-based mental training embedded into lessons (Khalaf et al., 2024) reflect high ecological validity and clearer operationalization of what athletes actually did. Conversely, where reporting was incomplete (Guo, 2014; Hökelmann et al., 2005) or where designs lacked robust comparators (Napolitano, 2017), it becomes difficult to isolate imagery-specific mechanisms, dose–response relationships, or the contribution of ancillary components (e.g., relaxation, self-talk, video modeling).

Another recurring theme is that multi-component psychological skills programs may produce broader or more reliable benefits than imagery alone, but they also complicate attribution. When imagery is delivered as one component of a package, performance gains may reflect synergistic effects (e.g., arousal regulation plus imagery), yet the independent contribution of imagery remains uncertain (Sharma, 2013). This is not a limitation per se for applied sport, but it matters for claims and for specifying minimal effective components.

From a coaching and applied sport psychology standpoint, the most defensible implication is that imagery is best positioned as a structured adjunct to physical practice, tailored to skill level and integrated into routine training cycles. The conditional effects by expertise and sequencing (Simonsmeier et al., 2018) suggest that novices may require preliminary emphasis on movement understanding, physical consistency, and basic imagery skill-building before imagery can reliably enhance performance. The lack of benefit from instruction matching and the observed performance decline across instruction phases (Veit et al., 2026) argue against over-prescriptive attentional cueing during execution. Thus, interventions should support functional, automatic control rather than induce reinvestment.

## 5 Conclusion

Overall, the available evidence indicates that imagery-based practice and broader psychological skills interventions may be associated with improvements in some gymnastics-related outcomes. However, certainty is low given that most controlled studies were rated some concerns/high risk and most non-randomized studies were rated serious/critical, alongside substantial heterogeneity in intervention content and outcome choice. For performance endpoints, several studies report benefits on judged skill/routine components or task-specific indices, but the evidence base does not yet support a claim of effectiveness due to design limitations, frequent non-blinding of judged outcomes, and inconsistent findings across comparators and expertise levels. For psychological/self-regulatory endpoints, imagery-based approaches more consistently relate to changes in variables such as confidence and perceived readiness, but these changes should be interpreted as proximal mechanisms or correlates rather than as proof of performance improvement. Accordingly, imagery should be viewed as a plausibly valuable, low-risk training adjunct that warrants further study, rather than as an evidence-established method. Higher-certainty, preregistered trials using competition-relevant outcomes and blinded external judging are required before any effectiveness recommendation is justified.

## Data Availability

The original contributions presented in the study are included in the article/Supplementary material, further inquiries can be directed to the corresponding author/s.
