# Supplementary material for: Mental imagery and performance in aerobic, artistic, acrobatics, trampoline and tumbling, and rhythmic gymnasts: a systematic review
Source: Front Psychol. 2026 Mar 11;17:1803261. doi: 10.3389/fpsyg.2026.1803261 (PMC13012994; doi:10.3389/fpsyg.2026.1803261)
Supplement: Supplementary file 1 [file Table_1.docx]

**Supplementary material**

**S1.** Main study design characteristics of the included studies.

| Study | Study design | FIG discipline | Apparatus event task | Competitive level | Sample (N) | Arms groups | Age (mean±SD) | Sex (n,%) | Primary performance outcomes measured | Secondary outcomes measured | Measurement timepoints |
| --- | --- | --- | --- | --- | --- | --- | --- | --- | --- | --- | --- |
| (Ahmed et al., 2021) | Quasi-experimental | Artistic (WAG/MAG) | Floor movements: wheel with quarter turn; back flip; back somersault straight | Club | 20 | Experimental (MI+skill training); Control (skill training) | 11–12 years | NR | Skill performance scores for the three floor skills | Mental imagery dimensions (visual, auditory, kinesthetic, emotional; overall) | Pre; Post (after 8 weeks) |
| (Battaglia et al., 2014) | RCT | Rhythmic | Vertical jump tests: Hopping Test (HT), Drop Jump (DJ), Counter Movement Jump (CMJ) | National | 72 | Experimental (video observation + PETTLEP imagery + physical practice); Control (physical practice + core/flexibility, no imagery/video) | Experimental: 13.8±1.3 y; Control: 14.2±1.7 y | Female: 72 (100%) | Jump performance: HT (Flight Time, Ground Contact Time); DJ (Flight Time, Ground Contact Time); CMJ (Flight Time) | Imagery ability (visual and kinesthetic) and correlations with post-training jump parameters (no results extracted here) | Baseline; Post (immediately after 6-week intervention; similar time of day) |
| (Calmels & Fournier, 2001) | Within-subject | Artistic (WAG/MAG) | Floor routine (individual competition-ready routines) | National | 12 | Within-subject: Mental execution condition; Physical execution condition | 16±2 y (13–20) | Female: 12 (100%) | Duration (s) of entire routine and of six routine stages under mental vs physical conditions; relative stage durations | Qualitative reports on imagery perspective and experience (informal interviews) | 3 trials per condition across 3 consecutive days (each day: mental + physical execution) |
| (Calmels et al., 2006) | Between-subject | Artistic (WAG/MAG) | Vault: Yurchenko vault (run-up, first flight, arm support, second flight) | National | 16 | Between-subject imagery perspective: Internal (FPP); External (TPP). Within-subject: Imagery vs Actual execution | 14.5±1.63 y (12–18) | Female: 16 (100%) | Chronometric outcomes: duration (s) of full vault and each of 4 stages during imagery and actual conditions across trials | Imagery process characteristics (ease, vividness, other modalities, emotions, self-talk) from post-experiment questionnaire | Pre (VMIQ 1 week before); Experimental session (imagery+actual, 3 paired trials); Post (manipulation check immediately after session) |
| (Di Rienzo et al., 2022) | Cross-sectional | Artistic (WAG/MAG) | Balance skill: modified arabesque on dominant leg (45° trunk inclination) + mental calculation control | Regional/state | 28 | Experts (gymnasts); Non-experts (non-gymnasts) | Experts: 20.15±3.62 y; Non-experts: 24.87±2.59 y | Women: 14 (50%); Men: 14 (50%) | Stabilometric (COP) variables during baseline, actual practice, VMI, KMI, and control. | Imagery ability (VMIQ-2) and perceived vividness ratings during VMI/KMI; subjective rating of arabesque performance. | Single session (baseline + randomized condition blocks); VMIQ-2 pre-test; vividness ratings after each VMI/KMI trial. |
| (Guo, 2014) | RCT | Aerobic | Aerobics routine: self-combination/group routine ('group 24×8' action sequence; assessed in standard game format) | Club | 30 | Experimental (imagery + conventional training); Control (conventional training only) | 16–19 years | Male and female (n, % NR) | Aerobics performance scores: Aerobic, Difficulty, Connection, Route, Music-with, Total | Imagery level dimensions (as above); training behavior/attitudes questionnaire (liking aerobics, willingness to spend more time/effort, clarity of goals) | Pre (baseline tests and imagery scale); Post (after 16 weeks) |
| (Hökelmann et al., 2005) | Quasi-experimental controlled pre–post | Rhythmic gymnastics | Jumps: Kosak jump; Grand Jete; jump with spread leg and ½ turn | NR | NR | Experimental group (mental training exercises + motor practice); Control group (comparison group; details NR) | NR | Female rhythmic gymnasts (n NR) | Movement quality of jumps (expert rating); mental representation performance on jump tests | NR (beyond cognitive representation and movement quality) | Pre-test; weekly testing across 4 weeks; post-test at end of 4 weeks |
| (Khalaf et al., 2024) | RCT | Artistic (WAG/MAG) | Roundoff + back handspring (connection); six performance stages A–F | NR | 62 | Control (instructional sessions without mental training); Experimental (instructional sessions + mental training) | 21.164±0.631 y (reported overall; range 21–22) | Male: 62 (100%) | Kinematic Coherence Scale scores for body-part coordination across six stages (A–F) of roundoff/back handspring performance | NR (no additional outcomes specified beyond kinematic coherence) | Pre; Post (after 7-week intervention) |
| (Marshall & Gibson, 2017) | Pilot randomized controlled trial; two-arm parallel groups with pre/post measures | Acrobatic gymnastics | Partnership competition routine (balance + dynamic elements + choreography); categories included WP, MxP, WG, MG | National level (competition partnerships) | 19 | Imagery training intervention; Control (usual training) | 13.2±2.7 years; inclusion age ≥9 | Male n=6; Female n=13 | Acrobatic performance score of competition routine | Competitive state anxiety (cognitive & somatic) and self-confidence; imagery use/functions | Baseline: pre-routine at practice competition. Post: after 4-week intervention, pre-routine at national championship competition (same routine). |
| (Napolitano, 2017) | Single-group case | Artistic (WAG/MAG) | Round-off flic (round-off + back handspring connection) | NR | 2 | Single-group case study (2 athletes); two-step protocol (questionnaire evaluation; video-based evaluation/prediction) | 12–15 years | NR | Round-off flic performance evaluation scores over time (judge/technician) and athlete self/hetero-evaluation scores | Awareness/ability to evaluate skill (agreement/consistency with judge ratings); prediction accuracy (forecast vs final outcomes) | Repeated monthly assessments across ~5 months (tables show 11 observations; described improvement at months 4–5) |
| (Nassib et al., 2022) | Crossover | Artistic (WAG/MAG) | Standing salto backward tucked (performed from standing; judged via FIG CoP criteria; recorded on force plate) | NR | 18 | Within-subject counterbalanced crossover: Visualization session; Mental imagery session (24 h apart) | 22.11±1.71 y | Male: 18 (100%) | Standing salto backward tucked performance: judged score (0–20) and force-plate outputs (jump/flight metrics from Quattro Jump) | Self-confidence; self-evaluation (self-rated performance). | Pre: imagery questionnaires (SIQ, VVIQ). Two sessions 24 h apart: each session baseline performance then post-strategy performance; SEQ completed at end; self-evaluation grid at end of experimental session(s). |
| (Rymal & Ste-Marie, 2017) | Quasi-experimental | Artistic (WAG/MAG) | Uneven bars routine (competitive routine; judges' execution score) | Regional/state | 18 | Between: FF-VSM+ATP; FF-VSM-only. Within: Competitions with FF-VSM vs without FF-VSM (2 of 4 competitions). | 9–16 y (Mage=12.11±2.17) | Female: 18 (100%) | Official judges’ uneven bars execution score (out of 10) at each competition | Self-regulatory processes/beliefs before/during/after bars performance (qualitative interviews); demographic/training variables | Four competitions across season (2 FF-VSM, 2 control; early vs late). Interviews immediately after one FF-VSM competition (n=10; 5 per group). |
| (Sharma, 2013) | RCT | Artistic (WAG/MAG) | Floor exercise routine | Club | 60 randomized; 52 completed | Experimental (PST + regular physical training); Control (regular physical training only) | 9–17 years | Randomized: male+female (n NR). Completed: Exp 12F/16M; Control 12F/12M (Total completed: 24F/28M) | Floor exercise performance score | NR (no additional outcomes reported beyond floor performance) | Pre (before PST); Post (after 6 weeks) |
| (Simonsmeier & Buecker, 2017) | Cross-sectional | Artistic (WAG/MAG) | Gymnastics competition performance across four disciplines (vault, bars, beam, floor) | Mixed | 75 | NR (observational; no intervention groups) | 7–16 y (M=10.87, SD=2.76) | NR | Competition performance percentage (mean across vault, bars, beam, floor) | Discipline-specific performance %; E-score and D-score (post hoc performance indices) | Questionnaires completed either 1 week before competition (at gym during training) or on competition day during warm-up; performance taken from the competition results in the year of data collection. |
| (Simonsmeier et al., 2018) | RCT | Artistic (WAG/MAG) | Uneven bars: cast to handstand | Mixed | 56 | Cross-over: imagery-first (imagery+regular then regular-only) vs imagery-last (regular-only then imagery+regular); expertise strata: low vs high | 7–15 y (M=9.63±2.43) | Female: 56 (100%) | Cast-to-handstand performance error score (0–7; lower=better) | Mental representation structure of the skill (SDA-M dendrograms; invariance λ; similarity to expert ARI); manipulation check responses; baseline imagery use/ability | T1 baseline (pre-phase 1); T2 (between phases after 4 weeks); T3 (post-phase 2 after 8 weeks total). Imagery use/ability assessed at T1 only; manipulation check after imagery phase (T2 for imagery-first; T3 for imagery-last). |
| (Veit et al., 2026) | RCT | Artistic (WAG/MAG) | Stretched jump with 450° longitudinal axis (LA) turn (jump test) | Regional/state | 29 | Between-subject instruction groups: External focus instruction; Internal focus instruction; Neutral instruction. Additional analytical grouping: Match vs No-match vs Neutral (based on VMIQ-2 vividness type). | 19.55±3.01 y (14–24) | Female: 29 (100%) | Jump performance variables: Jump height; LA deviation (LAD); hip angle; knee angle; body alignment (270° and 360°); leg/knee separation in flight; closed legs at landing; steps after landing. | Imagery vividness (VMIQ-2) and sociodemographic/sport-specific questionnaire data (age, expertise). | Online questionnaire prior to lab test. Lab session: baseline test (after warm-up and 1 trial: 6 jumps); intervention: 4 rounds × 5 jumps with instruction shown before each round (instruction read each round). |

**S2.** Characteristics of imagery procedures in the included studies.

| Study | Imagery type | Imagery content function | Perspective | Modality | Timing context | Dosage (len×freq×weeks) | Delivery format | Integration with practice | Co-interventions | Comparator control | Manipulation check or imagery ability measure |
| --- | --- | --- | --- | --- | --- | --- | --- | --- | --- | --- | --- |
| (Ahmed et al., 2021) | Mental perception training program / mental imagery accompanying skill training | Progressive muscle relaxation (Jacobson); basic mental imagery; multidimensional mental imagery; imagery of the studied floor skills | NR | Multisensory | During training | 15 min × 3/wk × 8 wk (24 sessions; total 360 min) | Coach-led | Interleaved | Ongoing skill training (both groups) | Control group: skill training only (no mental imagery program) | Martinez (1982) sport mental imagery scale (Arabized Ratib 2000; reformulated Jaber 2002); 4 dimensions: visual, auditory, kinesthetic, emotional |
| (Battaglia et al., 2014) | Video observation and PETTLEP mental training protocol | Imagery/observation of three vertical jumps (HT, DJ, CMJ) to enhance jumping performance; PETTLEP emphasis on task realism, real-time imagery, proprioceptive sensations and emotions; no relaxation phase described | Mixed | Visual+Kinesthetic | During training | 6 weeks; 2 sessions/day (each 3h) with 1 rest day/week; per session: 3min video observation + mental imagery (5 repetitions per jump; ~3min) + physical practice (5 repetitions per jump; ~3min) | Group session | Before physical practice | Both groups: leg strength/power/stiffness training and the same number of vertical jumps; diet/hydration monitored per FGI guidelines | Control group performed light core (abdominal) and active flexibility training immediately before the same physical practice; no self-directed imagery or video observation during intervention | MIQ-R at baseline (experimental group; completed twice on two days for reliability). Manipulation check interview: 10 open-ended questions by psychologist (vividness, controllability, kinesthesis, motivation, ease/speed, duration, involvement) |
| (Calmels & Fournier, 2001) | Mental execution / mental rehearsal of gymnastic routine | Imagined performing own floor routine in a competition situation; focus on temporal organization/speed of imagery | NR | NR | During training | 1 mental routine/day × 3 consecutive days (3 trials); plus 1 physical routine/day × 3 days | Self-guided | Stand-alone | NR (normal training ongoing; routines practiced daily under choreographer supervision) | Physical execution of the same floor routine performed as in competition | No standardized imagery ability measure; informal 5–10 min talks with 6 participants about perspective, ease, emotions, reasons (notes taken) |
| (Calmels et al., 2006) | Motor imagery of vault from first-person (internal) or third-person (external) visual perspective | Imagine performing own Yurchenko vault in preferred perspective, standing, with expectation of performing it physically afterwards; task divided into 4 stages with finger taps at stage transitions | Mixed | Visual | During training | Single experimental session: 3 imagery trials + 3 actual trials (each imagined trial immediately followed by actual trial); plus 5 practice attempts for finger-tapping system; VMIQ administered 1 week prior | Individual session | Before physical practice | NR (warm-up allowed as per normal routines prior to session) | Actual execution of the same vault (timed), and comparison between imagery perspectives (internal vs external) | VMIQ (French version) to determine preferred perspective + gymnast verbal reports + sport psychologist assessment; post-session manipulation check questionnaire/discussion covering ease, vividness, modalities, emotions, self-talk, and perspective consistency |
| (Di Rienzo et al., 2022) | Visual Motor Imagery (VMI) and Kinesthetic Motor Imagery (KMI) of the arabesque; assessed via stabilometry | Imagine performing the arabesque variation for 15 s while standing motionless (eyes open) vs physically performing the arabesque on one leg | NR | Visual+Kinesthetic | Other (specify) | Single laboratory session: Baseline stance 15 s (1 trial); Actual practice 15 s (4 trials); VMI 15 s (4 trials); KMI 15 s (4 trials); Control mental calculation 15 s (1 trial). Conditions administered in random order (block randomization) after baseline. | Individual session | Stand-alone | Control task: mental calculation during stance (15 s). | Between-groups (experts vs non-experts) and within-subject across conditions (Actual practice vs VMI vs KMI vs Control; baseline normalization). | VMIQ-2 (internal visual, external visual, kinesthetic; 12 items each modality) administered pre-testing; trial-by-trial vividness ratings after each VMI and KMI trial (Likert 1–6). |
| (Guo, 2014) | Imagery training / appearance training combined with conventional training | Visual imagery from demonstrations/videos/pictures + kinesthetic imagery during repeated practice; recall movements with eyes closed; integrated practice with video feedback and correction | NR | Visual+Kinesthetic | During training | 16 weeks (~4 months); Mon–Fri sessions reported (time window stated as '16:00 to 6:00'—likely typo/unclear); exact session duration NR | Coach-led | Interleaved | Both groups received the same conventional training content, schedule, environment, and equipment | Conventional aerobics routine training only | Movement Imagery Scale (name/version NR) at baseline; dimensions reported: stimulus special motivation, aroused motivation, excitation control motivation, special recognition, general cognitive |
| (Hökelmann et al., 2005) | Mental training exercises / mental representation (‘Gymnastic mental’) | Skill-specific mental representation of jumps; alignment/matching of images to strengthen cognitive component (temporal, spatial, kinaesthetic, linguo-symbolic elements) | NR | Predominantly visual (video + image alignment); kinaesthetic elements discussed but protocol specifics NR | NR (mental training integrated with motor learning; weekly tests) | 4 weeks; weekly tests; mental training dose per session NR | Computer-aided tool + video; participant aligns/matches images (individual delivery assumed; NR) | Mental and motor exercises executed holistically (combined approach stated) | Concurrent motor practice of jumps (details NR) | Control group (Ko-Gruppe); content not described (NR) | Author-developed mental representation tests within ‘Gymnastic mental’ (picture alignment task) administered pre/post and weekly |
| (Khalaf et al., 2024) | Seven mental training sessions (relaxation; controllable control; mental imagery for performance via video observation) | Mental training to build a clear mental image of the roundoff→back handspring motor sequence and reduce fear/negative self-talk; components: breathing-based relaxation, 'controlling the controllables' (attitude/effort/focus/preparation), and observation of correct skill sequence via video (regular + slow motion). | NR | Visual | During training | 14 min 30 s × 1/wk × 7 wk (total 101 min 30 s); delivered once per educational lesson after warm-up and before physical practice | Coach-led | Before physical practice | Both groups: 36-min physical warm-up + practice of roundoff/back handspring. Control: verbal/manual feedback and sometimes still images. Experimental: mental training (14:30) + physical practice (21:30) each lesson. | Control group completed warm-up then proceeded directly to physical practice (no mental training sessions). | NR (no imagery ability scale reported). |
| (Marshall & Gibson, 2017) | Imagery training intervention (script-based imagery) | Competition-routine rehearsal emphasizing calm-to-excitement arousal regulation and mastery/confidence; includes choreographic flow, skill execution cues, and positive outcome imagery | Internal / first-person (script written as 'you' experiencing routine) | Visual + kinesthetic; auditory (start beep, music, audience); emotional/arousal cues | Pre-performance/competition simulation; sessions conducted before physical training; imagery in real-time sequence of routine | 15 min × 2 sessions/week × 4 weeks (planned 6 weeks but reduced); total 8 sessions (~120 min) | Progressive, partnership-specific imagery scripts (see appendices) + imagery diary after each session | Imagery sessions completed before gymnasts’ physical training sessions | Usual acrobatic gymnastics training continued in both groups | Control group continued original training; no imagery training | Sport Imagery Questionnaire (SIQ) administered pre/post (imagery function/use assessment) |
| (Napolitano, 2017) | Motor imagery in first person and third person (mental simulation of round-off flic) | MI practice used over ~5 months to improve execution and evaluation awareness; athletes trained in both 1st- and 3rd-person MI and asked to evaluate own and others’ skill performances | Mixed | NR | During training | 5 months (frequency/session duration NR) | Coach-led | Interleaved | Normal technical training (implied); MI practiced alongside training (details NR) | No separate control group; comparisons between self/hetero ratings vs judge/technician ratings over time; pre–post within athletes implied | No standardized imagery measure reported; athletes educated on MI modalities (first- and third-person) prior to training |
| (Nassib et al., 2022) | Mental imagery (MI) and Visualization (video-based) | MI: 1-min mental simulation to improve standing salto backward tucked; individualized based on imagery questionnaires; allowed eyes open/closed; instructions included watching movement, feeling body in space, and listening to movement-related sound. Visualization: 1-min video of participant’s own baseline performance (front + profile, real-time + slow motion), external modality, no sound/text. | Mixed | Multisensory | During training | Acute: 2 sessions, 24 h apart. Each session: baseline trial + 1-min mental strategy (V or MI) + post-strategy trial. | Guided audio/script | Before physical practice | Standardized warm-up: 5 min moderate running + movement/specific muscular actions; standardized breakfast; controlled environment (23±1°C; 41±2% RH; 11:00–13:00). | Within-subject comparison: performance after MI vs after Visualization, with baseline (pre-strategy) performance within each session as covariate/control. | Sport Imagery Questionnaire (SIQ; Hall & Martin 1997) and Vividness of Visual Imagery Questionnaire (VVIQ/VVIQ2) completed pre-experiment; post-experiment interview on imagery nature; no trial-by-trial vividness rating reported. |
| (Rymal & Ste-Marie, 2017) | Feedforward video self-modeling (FF-VSM); advanced training program (ATP) on attention focus, imagery, goal setting, self-instruction | FF-VSM shows edited 'future' near-perfect uneven bars routine assembled from best skill executions; ATP trained athletes to integrate FF-VSM with attention focus, imagery (visual+kinesthetic; first/third person; timing control), goal setting (SMARTS), and self-instruction | External/third-person | Visual+Kinesthetic | Pre-competition | ATP: 4 weeks pre-season, 2 workshops/week (8 sessions total). Competitive-season FF-VSM: 2 of 4 competitions; per competition: 3 viewings ~5 min before bars warm-up + 1 viewing after warm-up (4 viewings/competition; total 8 viewings). Each viewing ~1.5–3 min depending on routine length. | Group session | Stand-alone | Routine training as usual; ATP group received additional workshops 1 month pre-season; both groups had FF-VSM videos constructed from training footage; videos updated during season when performance improvements occurred. | Control competitions: no FF-VSM (athletes followed typical precompetition routine). Between-groups: no-ATP FF-VSM group vs ATP+FF-VSM group. | MIQ-R (Hall & Martin, 1997) visual and kinesthetic imagery ability; administered during ATP Week 2 (ATP group) and 1 week pre-season (FF-VSM-only group). |
| (Sharma, 2013) | Psychological Skills Training (PST) program including imagery | Imagery taught/practiced as one component of a multi-skill PST package (goal-setting, relaxation, self-talk, imagery, attention/concentration, confidence); specific imagery scripts/content NR | NR | NR | During training | 30–45 min × 5/wk × 6 wk (PST sessions), alongside regular physical training | Coach-led | Interleaved | Regular physical training (both groups) | Control group received regular physical training only (no PST) | NR |
| (Simonsmeier & Buecker, 2017) | Imagery use (SIQ-C functions) and imagery ability (Sport Imagery Ability Questionnaire) | Measured imagery functions: cognitive specific, cognitive general, motivational specific, motivational general–affective, motivational general–mastery (SIQ-C); ability domains: skill, strategy, goal, affect, mastery (SIAQ). | NR | Multisensory | Pre-competition | NR (no imagery intervention; one-time questionnaire assessment per athlete) | NR | Stand-alone | NR | No control condition) | Imagery use: Sport Imagery Questionnaire–Children (SIQ-C, 21 items; 5-point scale). Imagery ability: Sport Imagery Ability Questionnaire (15 items; 5-point scale used in this study). |
| (Simonsmeier et al., 2018) | Audio-script guided motor imagery training (internal perspective; PETTLEP-informed) | Inner rehearsal of cast-to-handstand BACs with visual and kinesthetic cues; performed in regular environment/gear; speed manipulated (1 slow, 2 real-time); always internal perspective; diary/protocol to document distractions/difficulties | Internal/first-person | Visual+Kinesthetic | During training | Workshop 20 min at start of imagery phase; then 5 min/session × 4 sessions/wk × 4 wk. Per session: listen to full audio script once + imagine 3 times. Total imagery repetitions: 48 per athlete. | Guided audio/script | Interleaved | Regular physical practice of cast-to-handstand (same amount across weeks and phases) | Regular physical training only phase (no imagery). Cross-over within teams; counterbalanced order. | Baseline imagery ability: SIAQ (translated to German; child-appropriate language). Baseline imagery use: SIQ-C (translated). Post-manipulation check questionnaire (4 items) assessing perceived effectiveness, use frequency, enjoyment. |
| (Veit et al., 2026) | Imagery vividness/type (external–visual, internal–visual, kinaesthetic, or mixed) assessed by VMIQ-2; used to define match vs no-match with instruction focus | Participants imagined 13 motor tasks in three imagery types (EVI/IVI/KI) with VMIQ-2; one added item reflected the tested gymnastic jump. Imagery type used to classify athletes for instruction matching (match vs no-match). | Mixed | Visual+Kinesthetic | Other (specify) | No imagery intervention (one-time imagery vividness questionnaire; laboratory instruction intervention during single session) | NR | Stand-alone | Warm-up + one test trial; 10 s rest between jumps; no advice/feedback during trials. | Between-group comparison across instruction conditions; within-subject comparison baseline vs instruction phases (four rounds). | VMIQ-2 (German) + added gymnastics-specific item; FoA manipulation check after each block (0–4). |
